# Effect of Topically Administered Chitosan-*N*-acetylcysteine on Corneal Wound Healing in a Rabbit Model

**DOI:** 10.1155/2017/5192924

**Published:** 2017-06-12

**Authors:** Corinna Fischak, Robert Klaus, René M. Werkmeister, Christine Hohenadl, Martin Prinz, Leopold Schmetterer, Gerhard Garhöfer

**Affiliations:** ^1^Center for Medical Physics and Biomedical Engineering, Medical University Vienna, Vienna, Austria; ^2^Christian Doppler Laboratory for Ocular Effects of Thiomers, Vienna, Austria; ^3^Croma Pharma, Korneuburg, Austria; ^4^Singapore Eye Research Institute, Singapore; ^5^Lee Kong Chian School of Medicine, Nanyang Technological University, Singapore; ^6^Department of Clinical Pharmacology, Medical University Vienna, Vienna, Austria

## Abstract

**Purpose:**

The present study was performed to investigate the effect of topically administered chitosan-*N*-acetylcysteine (C-NAC) on corneal wound healing in a rabbit model.

**Methods:**

A total of 20 New Zealand White rabbits were included in the randomized, masked, placebo-controlled experiment. A monocular epithelial debridement was induced by manual scraping under general anesthesia. Animals were randomized to receive either C-NAC two times daily or placebo. Monitoring of corneal wound healing was performed with ultra-high-resolution optical coherence tomography (OCT) and epithelial fluorescein staining. Measurements were done immediately after and up to 72 hours after wound induction.

**Results:**

No difference in wound size was found immediately after surgical debridement between the C-NAC group and the placebo group. Wound healing was significantly faster in the C-NAC group compared to the placebo group (*p* < 0.01 for both methods). A good correlation was found between the OCT technique and the epithelial fluorescein staining in terms of wound size (*r* = 0.94).

**Conclusions:**

Administration of C-NAC containing eye drops twice daily leads to a faster corneal wound healing in a rabbit model of corneal debridement as compared to placebo. Ultra-high-resolution OCT is considered a noninvasive, dye-free alternative to conventional fluorescein staining in assessing corneal wound healing also in humans.

## 1. Background

Normal visual function is strongly dependent on an intact corneal surface [1]. Corneal epithelial defects are associated not only with reduced and/or blurred vision but also with ocular discomfort including pain, tearing, light sensitivity, eye redness, and foreign body sensation [1]. Causes for corneal epithelial defects include not only mechanical injuries, such as ocular surface traumas, ocular inflammatory diseases, and neurotrophic diseases, but also iatrogenic ophthalmic procedures.

To ensure fast wound healing after corneal injuries, the corneal epithelium has the ability to renew itself by a complex sequence of epithelial cell apoptosis, proliferation, migration, differentiation, and extracellular matrix remodeling [2]. Depending on the cause and the size of the area of the corneal damage, wound healing in humans may take up to 7 days, which corresponds to the reported turnover of the corneal epithelium [3, 4] Current treatment strategies for corneal epithelial defects include treatment with artificial tears and topical lubricants, frequently used in combination with topical antibiotics [5, 6]. Artificial tears and lubricants not only reduce the mechanical stress and therefore ocular discomfort but also dilute inflammatory cytokines, which in turn may be beneficial for corneal wound healing [1, 2, 7–9].

Because of their favorable biological properties, chitosan-based biopolymers have been proposed as scaffold materials for tissue regeneration and wound healing in several organs including the human eye. Among these, chitosan-*N*-acetylcysteine (C-NAC), a new biopolymer based on thiolated chitosan, has been recently introduced as a compound in topically administered eye drops (Lacrimera®) which received CE marking in Europe. Besides its good lubricant effect, two main advantages make C-NAC an interesting option for the treatment of corneal epithelial effects: First, the mucoadhesive effect of chitosan leads to a long residence time on the ocular surface [10]. Second, the introduction of thiol groups facilitates a chemical interaction of the polymer with ocular surface mucins. Thus, the resulting stabilization of the polymer-mucin network on the ocular surface may be beneficial for ocular wound healing [11, 12].

In the present randomized, masked, parallel group study, the effect of C-NAC on corneal wound healing was investigated in a rabbit model of corneal damage. To assess the corneal wound healing rate, two independent methods were used: ultra-high-resolution optical coherence tomography (OCT) and slit lamp photography of fluorescein-stained ocular epithelium.

## 2. Methods

### 2.1. Experimental Paradigm

Female New Zealand White rabbits (Charles River, Germany) weighing 2.3–4.1 kg were included in the experiments. This research followed the ARVO Statement for the Use of Animals in Ophthalmic and Vision Research and was approved by the local animal welfare committee. The animals were individually housed in an environmentally controlled room (12 : 12 h light : dark cycle, temperature 20°C, 60% relative humidity) at the Medical University Vienna, Department of Biomedical Research. Rabbits were fed with a commercial pelleted diet (ssniff K-H), and tap water was supplied ad libitum. Hay was provided as dietary supplementation. Rabbits were acclimated for a minimum of 14 d after arrival before any research manipulations were performed.

Surgical interventions were performed under general anesthesia using intramuscular injection of ketamin and midazolam. In addition, 0.16 ml/kg buprenorphin was administered for analgesia. To avoid pain during the wound healing period, 0.08 ml/kg carprofen was administered every 24 hours.

#### 2.1.1. Corneal Wound Healing Model

A circular debridement wound model in rabbits was used to investigate corneal wound healing. For this purpose, a corneal wound of 6 mm in diameter was introduced using a trephine to mark the wound area. Then the corneal epithelium was removed with a dull-bladed knife. Care was taken to avoid damage of deeper corneal layers.

### 2.2. Chitosan-N-acetylcysteine

C-NAC is based on a biopolymeric chitosan backbone, which has been derivatized with thiol groups by the introduction of *N*-acetylcysteine. C-NAC eye drops in its final formulation contain 0.1% of the thiolated polymer in a physiologically buffered solution with a mildly acidic pH. The formulation is CE-marked as a medical device under the trade name Lacrimera (Croma Pharma GmbH, Leobendorf, Austria). Safety and efficacy of the product are well described and were shown to be excellent in a recently published controlled, randomized double-masked clinical study [13].

#### 2.2.1. Study Design

The present study was performed in a randomized, controlled, masked, parallel-group design. After induction of the corneal wound, OCT measurements were performed to assess baseline wound size. In case OCT analysis revealed damage of the corneal stroma, the animal was excluded from the trial. Then fluorescein staining was performed, and photographs of the corneal epithelium were taken to assess the wound size. After completion of baseline measurements, either 50 *μ*l of placebo (phosphate-buffered saline solution) or C-NAC eye drops were instilled on the ocular surface twice daily. Measurements of corneal wound healing using OCT and fluorescein staining were repeated 12, 24, 36, 48, and 72 hours after corneal wound induction.

#### 2.2.2. Methods

*(1) Assessment of Corneal Wound Healing Using Ultra-High-Resolution OCT*. Corneal wound healing was assessed with a custom-built ultra-high-resolution OCT system as described previously [14] differing only in the light source used. For technical reasons, the system used in the current study is based on a superluminescent diode (SLD) with a central wavelength of 850 nm and a spectral bandwidth of 165 nm. The theoretical axial resolution of the device is 1.3 *μ*m in corneal tissue, while the lateral resolution given by the focusing optics is approximately 18 *μ*m. The incident power of the probe beam onto the cornea was set to 2.5 mW for acquisition of the corneal volumes in order to measure the epithelial wound. This value is well below the maximum permissible exposure as specified by ANSI [15] and IEC 60825–1 [16].

For evaluation of corneal wound healing, one OCT volume with a size of 7.5 × 7.5 × 1 mm (horizontal × vertical × depth) and comprising 1024 × 512 × 1024 pixels was recorded within 5 seconds. After the first postprocessing steps including rescaling and dispersion compensation, the acquired volumes were resliced in axial direction in order to obtain an en face image of the anterior cornea. The borders of the corneal erosion were segmented using custom software written in LabVIEW (LabVIEW 2013, National Instruments, Austin, TX, USA). To obtain an absolute measure for the wound area, firstly, the scanning range of the OCT system was taken into account. In the second step, the distortion of the en face image due to the curvature of the cornea was corrected.

*(2) Assessment of Corneal Wound Healing Using Fluorescein Staining*. Fluorescein drops (Minims-Fluorescein Sodium 2.0%; Chauvin Pharmaceuticals Ltd.) were instilled into the study eye, and photographs were obtained under illumination with cobalt-blue light using a digital camera. To calibrate each image for the calculation of absolute diameters, a ruler was placed near the eye and photographed together with the cornea. The area of corneal abrasion was measured semiautomatically with a custom macro written for ImageJ (National Institutes of Health, Bethesda, MD; available in the public domain at http://rsbweb.nih.gov/ij/). Measurements were corrected separately for each image using a calibration factor based on the photographs of the ruler.

#### 2.2.3. Data Analysis

To detect differences in the time course between the two treatment groups, a repeated measures ANOVA model was applied. To calculate the correlation between the two different measurement techniques, Pearson's correlation analysis was performed. All statistical analyses were carried out using CSS Statistica (Version 6.0, Tulsa, Oklahoma, US).

## 3. Results

A total of 20 animals were included in the experiment. In the placebo group, baseline defect areas were 24.6 mm^2^ ± 3.1 mm^2^ as measured using OCT and 31.4 mm^2^ ± 5.9 mm^2^ as assessed by fluorescein staining, respectively. In the C-NAC group, baseline areas of corneal debridement were 27.4 mm^2^ ± 5.5 mm^2^ as measured with OCT and 32.8 mm^2^ ± 4.8 mm^2^ as assessed with fluorescein staining. No statistical significant difference was observed in baseline corneal wound size between the C-NAC and the placebo group with neither of the techniques (OCT: *p* = 0.2 between groups, fluorescein staining: *p* = 0.6 between groups).

**Figure 1:**
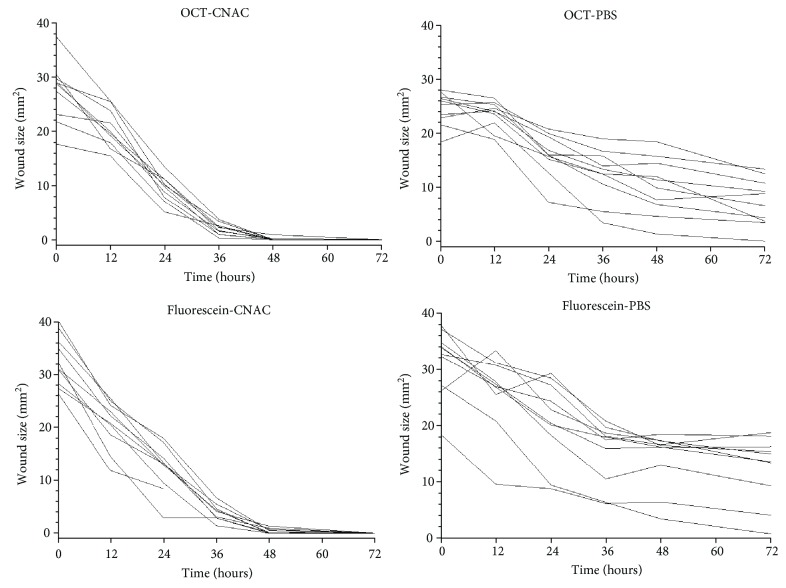
Area of defect over time after treatment with either placebo or C-NAC. Data are presented individually based on either fluorescein staining of corneal epithelium or optical coherence tomography.

**Figure 2:**
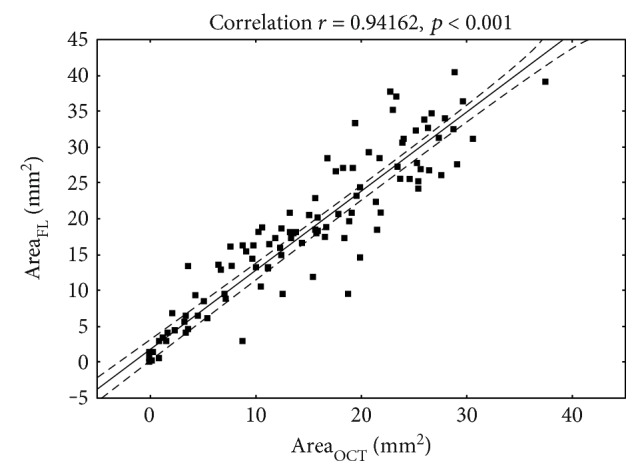
Correlation between areas of defect as measured by photography after fluorescein staining or optical coherence tomography.

As shown in Figure 1, wound healing over time was faster in the C-NAC group compared to the placebo group. This was statistically significantly both for the OCT-measured defect size (*p* < 0.01, ANOVA time versus treatment) and for the fluorescein-measured defect size (*p* < 0.01, ANOVA time versus treatment). As shown in Figure 2, a good correlation was observed between the two measurement techniques in assessing the size of corneal defect (*r* = 0.94, *p* < 0.05).

## 4. Discussion

The data of the present study indicate that corneal wound healing in a rabbit model of epithelial damage was significantly improved when C-NAC containing eye drops were applied compared to the placebo treatment. In addition, it appears that high-resolution OCT is a suitable technique for the noninvasive measurement of corneal epithelial damage in the rabbit model of corneal debridement. This is in agreement with a recent study in humans where ultra-high-resolution OCT was used to quantify epithelial defects in keratoconus patients undergoing standard collagen cross-linking [17].

Besides their valuable role in the treatment of dry eye disease, it is known that topical lubricants containing, for example, hydrating polymers such as hyaluronic acid (HA) or hydroxypropyl methylcellulose (HPMC), may enhance corneal reepithelialization and wound healing [7–9]. The data of the current study supports the concept that this holds also true for C-NAC. The underlying mechanism is not entirely clear but may be at least partially related to the effects of unmodified chitosan on wound healing [17, 18]. It has been shown in previous studies that in a rabbit alkali burn model, chitosan reduces scar formation and increases the collagen density in the conjunctiva [19]. Further evidence shows that chitosan leads to an increased growth and proliferation of cultured corneal epithelial cells in vitro [20]. This effect has been mainly attributed to increased epithelial proliferation and migration induced via the extracellular signal-regulated kinase (ERK) pathway [21]. This may well be the mechanism that underlies increased corneal wound healing of C-NAC used in the study, although this hypothesis requires further investigation.

In addition, the specific chemical and biological properties of C-NAC may contribute to the observed beneficial effect on corneal wound healing. Chemically spoken, C-NAC is a positively charged macromolecule with chitosan as a polycationic polysaccharide backbone. Chitosan is further modified by the introduction of *N*-acetylcysteine (NAC) via nucleophilic substitution and by the introduction of thiol groups. The latter modification determines to a large degree the biological properties of C-NAC on the ocular surface [22]. Via the introduced thiol groups, C-NAC binds electrostatically and chemically to negatively charged reactive mucins of the ocular surface, thereby producing a stable glycocalyx-like structure. These scaffolding properties of C-NAC may lead to an enhanced stability of the polymer-mucin network, resulting in a prolonged residence time [23, 24] and promoting corneal wound healing.

Prolonged ocular residence time of C-NAC in comparison to nonmodified chitosan was actually demonstrated in preclinical studies in a rabbit in vivo model using microPET technology [10]. The results show that C-NAC can be detected on the ocular surface for up to 48 hours [25] after application, an effect considerably longer than with unmodified chitosan.

The use of two independent methods for the assessment of corneal wound healing strengthens the data obtained in the present study. Classically, the measurement of lesion size is performed using fluorescein staining of the corneal defect followed by the measurement of wound size by means of slit lamp examination or photography [26–29]. This approach is however limited by the need for a dye to perform corneal staining, which in turn may influence wound healing. Further, the measurement of wound size itself is challenging, because it is dependent on complex optical properties such as the distance to the measured eye, refractive error of the examiner, and the magnification of the imaging device. To compensate for these limitations, we graded the corneal defect size based on calibrated photographical images of the ocular surface.

As a more sophisticated technique to measure epithelial defect size, a custom built ultra-high-resolution OCT system was used. The technical details of this device have been described in detail previously [14]. We have used this system in several human studies to assess precorneal tear film thickness [14, 30–32] as well as to measure corneal wound healing in humans (Bata et al. 2016 JAMA Ophthalmology, in press). The latter study shows that in humans, there is a good correlation between corneal wound size measured with OCT and measurements based on corneal fluorescein staining [17].

This finding is in agreement with the results of the current experiment (Figure 2). However, it was noted that the erosion area determined via OCT measurement was slightly smaller than the wound area determined from fluorescein images. This might be related to the different methods used for detection of the borders of the corneal defect. Thus, OCT detects the structural border of the epithelial wound based on differences in reflectivity of the different tissues. Fluorescein images, on the other hand, rely on an “indirect” visualization of the erosion boundary by detection of fluorescence light arising from new epithelial foci. This signal however is influenced by different factors, such as ambient light, amount of stimulation light, and dynamic range of the camera in use; slight variations of these parameters might lead to an overestimation of the real erosion size.

Our study also had some limitations that are worth to be mentioned. As topically applied agents may influence corneal wound healing per se, no generally accepted placebo control exists for studies on ocular surface disease. In the present study, PBS eye drops have been used twice daily to guarantee investigator masked conditions during the study. It can, however, not be excluded that PBS had a beneficial effect on corneal wound healing due to its lubrication effects leading to an underestimation of the effect of C-NAC. Further, it is not entirely clear to what extent data gained in rabbit experiments reflect the situation of corneal wound healing in humans. However, because of their considerable advantages in comparison to other animal models, rabbit models of corneal healing have been widely used in the past to assess reepithelialization and scarring. It has been shown that the rabbit cornea responds to a large degree comparable to the human cornea in respect to wound healing time, the extent of scarring, and myofibroblast formation [33, 34]. In addition, the rabbit cornea is similar in size to the human cornea, which confirms its suitability of the model for translational research. [33].

In conclusion, our data show that C-NAC containing eye drops improve corneal wound healing in a rabbit model of corneal epithelial debridement and may therefore also be considered beneficial for the treatment of human corneal epithelial defects. In addition, we have demonstrated that ultra-high-resolution OCT is an excellent, noninvasive, dye-free technique for the monitoring of corneal epithelial healing in both humans and experimental animals.
